# Venom Variation during Prey Capture by the Cone Snail, *Conus textile*

**DOI:** 10.1371/journal.pone.0098991

**Published:** 2014-06-18

**Authors:** Cecilia A. Prator, Kellee M. Murayama, Joseph R. Schulz

**Affiliations:** Department of Biology, Occidental College, Los Angeles, California, United States of America; Swiss Institute of Bioinformatics, Switzerland

## Abstract

Observations of the mollusc-hunting cone snail *Conus textile* during feeding reveal that prey are often stung multiple times in succession. While studies on the venom peptides injected by fish-hunting cone snails have become common, these approaches have not been widely applied to the analysis of the injected venoms from mollusc-hunters. We have successfully obtained multiple injected venom samples from *C. textile* individuals, allowing us to investigate venom compositional variation during prey capture. Our studies indicate that *C. textile* individuals alter the composition of prey-injected venom peptides during single feeding events. The qualitative results obtained by MALDI-ToF mass spectrometry are mirrored by quantitative changes in venom composition observed by reverse-phase high performance liquid chromatography. While it is unclear why mollusc-hunting cone snails inject prey multiple times prior to engulfment, our study establishes for the first time a link between this behavior and compositional changes of the venom during prey capture. Changes in venom composition during hunting may represent a multi-step strategy utilized by these venomous animals to slow and incapacitate prey prior to engulfment.

## Introduction

During the last 50 million years, cone snails (genus *Conus*) have evolved into hundreds of species with prey preferences that generally fall within three categories: fish-hunters, worm-hunters, and mollusc-hunters [1]. It is widely accepted that each feeding group, if not each species, has its own set of unique venom profiles used to target the species' prey [2]. Interspecies venom variation is also well established and recent studies have demonstrated that intraspecific variation exists in the composition of prey-injected venom peptides from several fish-hunting species [3]–[6]. These instances of variation might be explained by stochastic, genetic as well as individual differences in age, size, or geographic location. Other venomous animals such as snakes and scorpions have been shown to exhibit variation due to diet or hunting strategy [7]–[10]. Studying venom variation, and the processes that regulate it, could aid in tapping into the vast amount of venom peptide diversity that has evolved within the genus *Conus*. While studies on injected venom have become more common in fish-hunting cone snails, this approach has not been widely applied to the analysis of venoms from mollusc-hunters; with the recent first analysis of pooled prey-injected venom samples from a mollusc-hunter, *C. marmoreus* [11]. Studies on the fish-hunter, *Conus striatus,* indicate that venom analyzed from the dissected duct is more complex than the injected venom from the same animal, revealing that mechanisms for venom peptide sorting, packaging, and/or selective delivery into prey must exist [5], [12].

Cone snails employ a distensible proboscis to hydraulically propel a hollow radular tooth into prey, serving as a conduit for the passage of venom [13]. Observations of the mollusc-hunting cone snails during feeding reveal that prey are often injected multiple times in succession [14]. During prey capture by the mollusc-hunting cone snail, *Conus textile*, the prey is typically not completely stunned or killed by a first venom injection and requires multiple injections before engulfment commences. It is common to see a prey item become stunned for a short period of time following the first injection of venom and then recover coordination and motility. The recovery of prey from paralysis is not an issue for many worm-hunting and fish-hunting species that inject prey only once before engulfment; the barbed radular tooth remains affixed to the stung prey and tethered to the snail [15]–[16]. Species that sting prey multiple times during a single feeding event may have compositional changes in the venom they inject that may allow them to slow, and finally, subdue prey. Recent proteomic studies of the venom peptides dissected from *C. textile* provide useful data sets for comparison with peptides found in the injected venom samples [17], [18]. However, previous analyses of *C. textile* venom have been limited to dissected venom samples and, although useful for peptide characterization, do not provide a clear picture of the functionally important peptides actively used to subdue prey.

In this study we report the first analysis of injected venom profiles from *C. textile*. While we identified intraspecific variation in the injected venom profiles of *C. textile*, a shared set of peptides was common among the first injections made by individuals during prey capture. However, we have identified qualitative as well as quantitative changes in the venom profiles from multiple injections by *C. textile* individuals during single feeding events. These changes in venom composition indicate that this venomous animal can regulate the delivery of venom components as part of its prey-capture strategy.

## Materials and Methods

### Experimental animals

This study was conducted in compliance with the Occidental College Institutional Animal Care and Use Committee for the use of non-vertebrate animals. Adult specimens of Fijian *Conus textile* (Linné 1758) were 52–60 mm shell length (Quality Marine, Los Angeles, CA). Animals were maintained in a 10-gallon saltwater tank at Occidental College, Los Angeles and fed live *Nassarius* species (<30 mm; Nautilus Tropical, Tampa, FL) once per week. Three individuals were labeled *i-iii* to track over the length of the experiment.

### Venom collection

Dissected pieces of *Nassarius* foot were sliced thinly and attached to the top of a small collection tube covered with a thin layer of latex to avoid water leakage. *C. textile* specimens were induced to inject venom into small collection tubes. To collect a sequential series (set) of venom injections, several collection tubes were prepared ahead of time. After venom collection, injected venom samples (9–10 µl) were centrifuged for 1 min and stored at −80°C. Injected venom samples were obtained from *C. textile i*–*iii*, representing the first injection. Additionally, two sets of venom injections were collected from *C. textile* specimens *i and ii.* Two injected venom samples (*C. textile i*, Set 1, second injection and *C. textile ii*, Set 2, third injection) were not successfully analyzed by reverse-phase HPLC (see below). Also, two venom injections were not successfully collected into the sample tubes (*C. textile ii*, Set 1, third injection and *C. textile ii*, Set 2, first injection).

### Mass spectrometry

To prepare venom samples for matrix-assisted laser desorption/ionization time of flight mass spectrometry (MALDI-ToF MS), injected venom was thawed and then re-centrifuged to separate venom granules from the supernatant. 1 µl of venom supernatant was diluted 1000-fold in a 50% acetonitrile (ACN), 0.1% trifluoroacetic acid (TFA) solution. 1 µl of the 1000-fold diluted sample was combined with 1 µl of matrix α-cyano-4-hydroxycinnamic acid. Averaged spectra were obtained in linear and reflectron modes with an Applied Biosystems Voyager DE-PRO MALDI ToF-MS (fitted with a 20-Hz nitrogen laser). For peak counts and comparisons, detection was limited to peaks greater than 10% of the spectral maximum intensity. All spectra presented were normalized to the maximum peak height. Several mass matches to known peptides were identified including matches made to calculated average masses from sequence data:

Genbank accession # FB299894: 1658.92 GCCSRPPCIANNPDLCG

Genbank accession # CAR81520: 2041.41 GCCSNPPCIAKNPHMCGGRR

### HPLC

**Figure 1:**
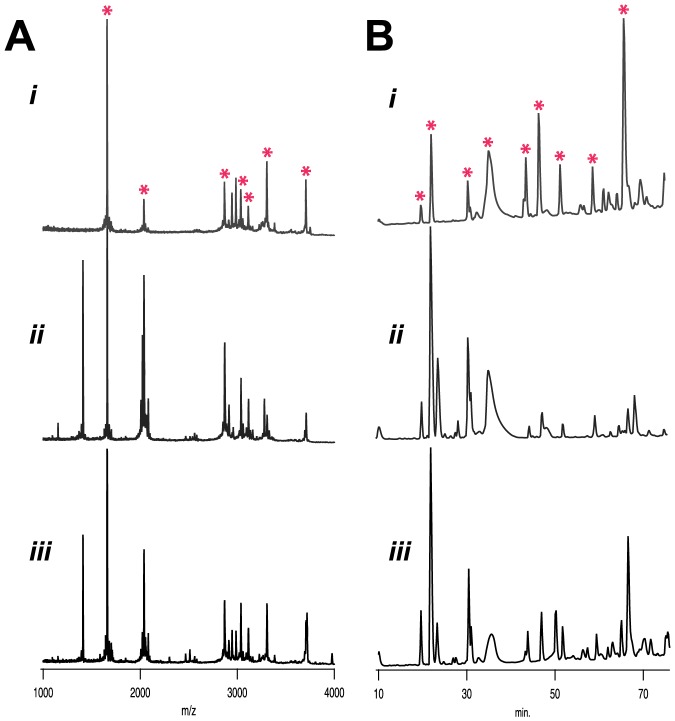
Analysis of intraspecific variation in 1^st^ injections from *C. textile*. (A) MALDI-ToF fingerprinting of 1^st^ injections from *C. textile i-iii* reveal a common profile of venom peptides marked by instances of variation between individuals. (B) Quantitative differences in venom composition shown by RP-HPLC. Chromatograms indicate more dramatic variation in quantitative differences between *C. textile* specimens. Peaks common to all three specimens are marked with asterisks. Chromatograms were normalized to maximum peak height.

Injected *C. textile* venom (cleared of granules as above) was suspended in a 0.1% TFA, 5% ACN solution and re-centrifuged. Venom (8 µl) was loaded on a C18 peptide trap cartridge (Michrom Bioresources) in-line with the sample loop and fractionated using an analytical reverse-phase HPLC column (RP-HPLC; Grace Vydac 218TP C18). Peptide elutions (monitored by absorbance at 214 nm) were achieved using a gradient of 5–80% B in A over 80 min at a flow rate of 0.5 ml/min. Solvent A was 0.1% aqueous TFA, solvent B was 100% ACN with 0.8% TFA. Organic solvents were removed from the LC fractions with a SpeedVac, and samples were stored at −80°C. Chromatogram comparisons were limited to peaks greater than or equal to 10% of the maximum peak height (see Figure 1).

## Results

### Analysis of injected venom from a mollusc-hunter

Venom profiles from the first injections made by three *Conus textile* individuals (labeled *i*–*iii*) were obtained by linear MALDI ToF mass spectrometry (figure 1A). Mass spectra obtained by MALDI ToF MS provide a qualitative “fingerprint” of individual injected venom profiles [5]. Intraspecific venom variation exists in this species, as first injections from each specimen revealed a set pattern of prominent venom peptides marked by instances of variation. Occurrences of a common set of peptides have also been observed in the fish-hunting cones [5], [6]. The results obtained by MALDI-ToF MS are mirrored by quantitative differences in venom composition profiles obtained by RP-HPLC of single injected venom samples (Figure 1B). These initial observations of injected venom from a mollusc-hunter reveal venom profiles to be more complex than those obtained from fish-hunting cone snails [4]–[6], [19], [20], suggesting the need for a more complex complement of peptides to subdue mollusc prey.

### Venom variation during prey capture

**Figure 2:**
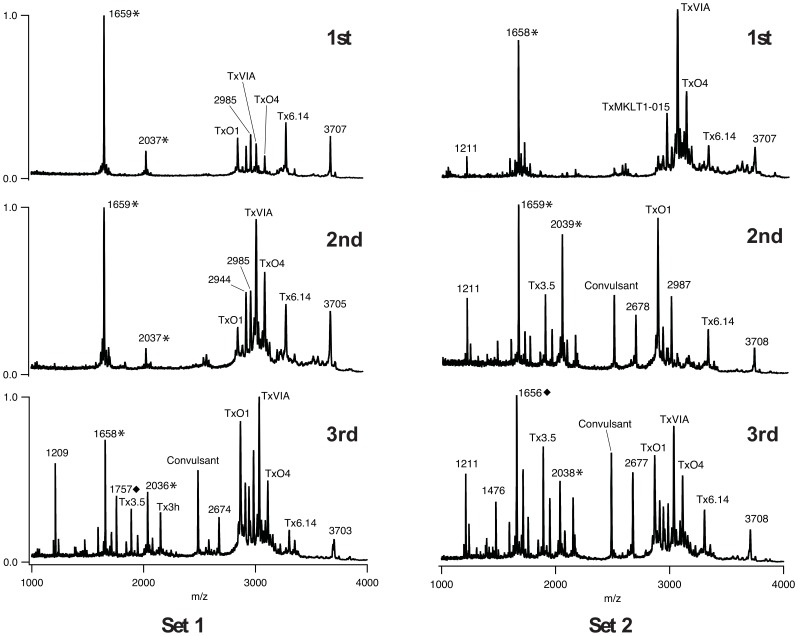
Mass spectra of the 1^st^, 2^nd^, and 3^rd^ injections from *C. textile i* for two prey capture events. In both sets, venom peptide variation was observed in subsequent injections and increased in complexity. Comparisons between the two prey capture events revealed variation between Sets 1 and 2 within the same individual (*i*). Sets 1 and 2 were collected 53 days apart. * match made to calculated average mass from sequence data as described in the methods section. ♦ mass similar to a peptide mass observed in a previous study [29].

**Figure 3:**
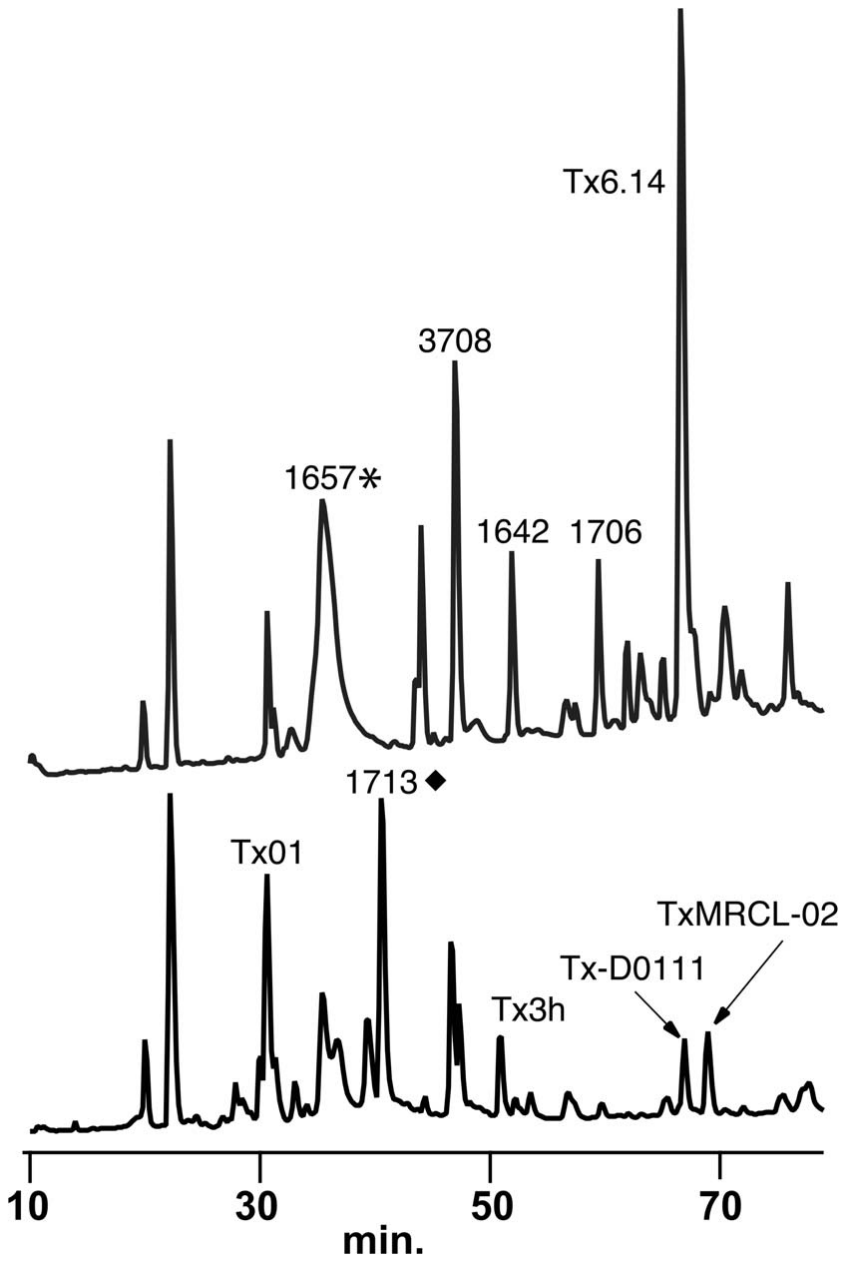
RP-HPLC chromatograms of 1^st^ and 3^rd^ injections from *C. textile i*. Quantitative differences were observed after multiple injections during a single prey capture event. Upper trace 1^st^ injection, lower trace 3^rd^ injection. Refer to figure 2 for symbol legend. Absorbance monitored at 214 nm. Chromatograms were normalized to the threshold peak (retention time 19.9 min.) utilized for the analysis presented in table S1.

To observe the possibility of compositional changes during single prey capture events, sets of injected venom samples were obtained from *C. textile* specimens *i* and *ii* (Movie S1). MALDI profiles revealed venom peptide variation between injections with increasing complexity of peptides detected by the third injection as compared to the first one. For example, in *C. textile i*, Set 2 increases in complexity by the second injection (Figure 2). On average there was a 138% increase in number of peaks detected (Set 1 peak counts increased from 9 to 26, Set 2 peak counts increased from 15 to 28 from first to third injections, respectively). In *C. textile ii, Set 2*, a clear first injection was made by the specimen, marked by the observation of a cloudy puff of venom, but the radular tooth did not pierce the latex of the collection tube, precluding analysis. Unique to Set 2 however, was the collection of a fourth injection. This fourth injection differed noticeably from the second and third injections and extends the trend of variation from early to late injections during a single feeding. Comparisons between the two individuals revealed that there is also some variation between each separate set of feeding events (Figures 2, 3). The majority of peptides from first, second, and third injections observed in MALDI profiles were previously described in dissected duct venom [18]. Peptide masses not matching those identified in dissected venom suggest the presence of novel venom components not previously described (Table 1).

**Table 1:** Peptides detected by MALDI-ToF MS.

	A Superfamily		M Superfamily					T Superfamily
Individual	1658✶	2037✶	1655 (Tx3a)	1712♦		1889 (Tx3.5)	2150 (Tx3h)	1475♦
*i*	1, 2, 3	1, 2, 3	**3**	ND		**2, 3**	**3**	**3**
*ii*	1, 2, 4	1, 2, 3, 4	**4**	**2, 3, 4**		**2, 3**	**2, 3, 4**	**3, 4**
*iii*	1, 2, 3	1, 2, 3	ND	ND		ND	ND	ND
	O Superfamily							
	**2487 (Convulsant)**		**2867 (TxO1)**	**2944 (TxMKLTI-015)**		**3035 (TxVIA)**	**3113 (TxO4)**	**3305 (Tx6.14)**
*i*	**2, 3**		1, 2, 3	1, 2		1, 2, 3	1, 2, 3	1, 2, 3
*ii*	**2, 3, 4**		1, 2, 3, 4	ND		1, 2	1, 2	1, 2
*iii*	ND		1, 2, 3	ND		1, 3	**2**	1, 2, 3
	Superfamily unknown							
	**1210**	**1408**	**2676**	**2910**	**2986**	**3132**	**3274**	**3707**
*i*	1, 2, 3	ND	**2, 3**	ND	1, 2	ND	ND	1, 2, 3
*ii*	**2, 3, 4**	1, 2	ND	**3, 4**	ND	**2, 3, 4**	1, 2	1, 2
*iii*	ND	1, 2, 3	ND	ND	ND	ND	ND	1, 2, 3

Peptides detected in the 1^st^, 2^nd^, 3^rd^ and 4^th^ injections (1,2,3,4) made by C. textile individuals *i-iii*. Masses matching known peptides or sequences in the database are listed by superfamily. Masses lacking a match to a known peptide or database sequence (Superfamily unknown) are listed as average masses. The 4^th^ injected venom sample corresponds with the sample collected only from *C. textile ii*. Injection numbers in bold were observed in 2^nd^ injections or later. Refer to Figure 2 for symbol legend. ND: not detected in the individual listed.

**Figure 4:**
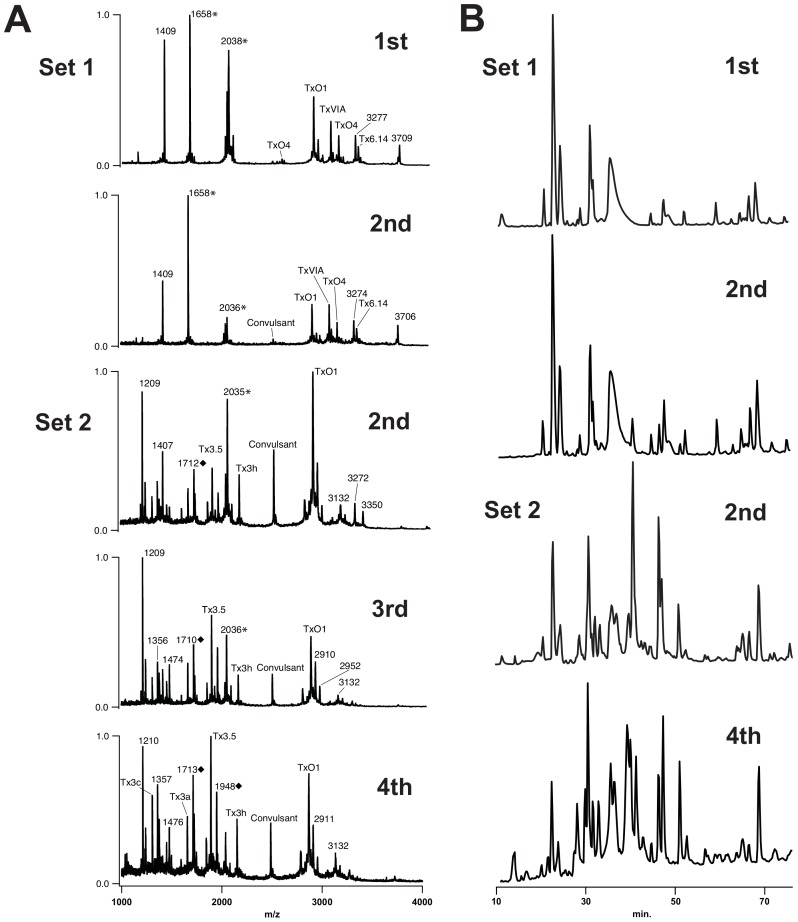
Comparison of injected venom samples from separate feeding events in *C. textile*. (A) Spectra from two separate prey capture events 243 days apart, including a 4^th^ injection from *C. textile* individual *ii.* (B) Corresponding RP-HPLC chromatograms illustrate quantitative differences observed within single prey capture events and between separate prey capture events in *C. textile ii.* Refer to figure 2 for symbol legend. Absorbance monitored at 214 nm. Chromatograms were normalized to maximum peak height.

MALDI profiles correlate with quantitative differences observed by RP-HPLC (Figures 3B, 4). For example, analysis of the first and third injections from *C. textile i,* Set 1, by RP-HPLC demonstrates quantitative differences in the abundance of injected venom peptides from the same feeding event (Figure 3). The identification of several major venom peptides that differed between injections (Table S1) was made by analyzing fractions with MALDI ToF MS.

An additional set of venom injections was obtained from a larger Hawaiian specimen (with larger volumes of venom) and compared to *C. textile* venom isolated directly from the venom duct (Figure S1). As expected, the venom isolated directly from the duct has the greatest complexity, even in the absence of extensive peptide extraction. The prey-injected venom profiles from this specimen are similar to those of the Fijian snails used in this study, with changes in composition in the 2^nd^ and 3^rd^ injections. Qualitative as well as quantitative (table S1) changes in venom peptide composition during prey capture are, therefore, a consistent feature of the envenomation process in *C. textile* and are not dependent on geographic distribution or the volume of venom injected.

## Discussion

We have identified both qualitative and quantitative changes in venom peptide composition during single feeding events of the mollusc-hunting cone snail, *C. textile*. It is not clear why mollusc-hunters inject prey multiple times prior to engulfment and how this relates to the observed changes in venom composition. The first injections from all *C. textile* specimens analyzed exhibited similar profiles when analyzed by MALDI profiling and, while some intraspecific variation was noted, a shared set of venom peptides was common among individuals (Figure 1, Figure S1 & Table 1).

Our observations of prey capture indicate that the first injection of venom does not immediately kill the prey. The first injection may be used to slow prey or modulate some aspect of the prey's physiology allowing subsequent venom peptides to have greater efficacy. As molluscs are a relatively slow-moving prey, it may be more efficient to inject a set of peptides to initially slow the prey followed by subsequent injections causing more complete paralysis. If a first injection subdues prey, a second and third injection may be required, with different venom peptides, to eventually kill the prey or, possibly, aid in its digestion. Also, the anatomy of a mollusc may not allow for the rapid paralysis seen in fish stung by cone snails. For fish-hunting cone snails, venom peptides that paralyze prey quickly are required to successfully capture fish capable of extremely rapid escape responses [21]. Future studies on the physiological effects and molecular targets of *C. textile* venom peptides may reveal the role of the various venom peptides injected during the first versus later injections. Unlike the prevenom observed in scorpions [9], the injected venom of *C. textile* does not change visibly to the eye in composition or significantly in measured volume during subsequent injections; the changes are in the relative abundance of the various venom peptides present as the injections continue.

Comparisons between the injected venom peptides observed in our MALDI profiles and those previously identified in the venom duct suggest which known classes of venom peptides are being utilized during prey capture (Table 1). We found that peptides from the O-superfamily were prominent in all venom profiles observed and were present in multiple shots from single feeding events, supporting the importance of O-superfamily members throughout prey capture. These peptides serve to block calcium and potassium channels or slow sodium channel inactivation [22]. Peptides of the M- and T-superfamilies, however, were more prevalent in subsequent injections (Table 1). Members of the T-superfamily block noradrenaline transporters or voltage-gated ion channels [23], [24]. While venom peptides from the M-superfamily are known blockers of either sodium channels, potassium channels or acetylcholine receptors [25], the peptides identified in the venom belong specifically to the mini-M branch of the superfamily and with potentially novel molecular targets [26]. These data provide evidence for a shift in venom peptide superfamily usage during feeding. Peptide masses not previously detected in the venom from dissected venom ducts indicate the presence of novel peptides and highlight the benefits of studying injected venom.

Cone snail venom peptides are expressed in a long tubular venom duct. Previous studies on dissected *C*. *textile* venom ducts indicate that venom peptides are secreted in specific regions along the length of the duct [17], [18], [27] which may explain the compositional differences noted in multiple injections during single feeding events. Studies on the biomechanics of prey capture in the mollusc-hunter, *Conus pennaceus*, indicate that discrete amounts of venom are injected into prey [28]. First injections might be more prominent in peptides found in regions of the duct proximal to its insertion into the mid-esophagus and later injections composed of peptides found in more distal regions near a large muscular venom bulb connected to the end of the duct. Our observations of the presence of the M-superfamily in later injections (see above) and their expression specifically in regions of the duct closer to the venom bulb [17], [27], distant from the mid-esophagus, support this model. The variation in peptide composition along the length of the venom duct may, therefore, have functional significance for the feeding behavior of these animals.

Our analyses of injected venom from a mollusc-hunting cone snail establish that, along with an elaborate venom injection system [13], [28], the composition of prey-injected peptides changes during single feeding events. Further investigations into the physiological effects of the various peptides on their diverse prey, their detailed expression patterns within the venom duct and the biomechanics of venom delivery will help to complete the mechanistic picture of prey capture. In addition to intraspecific venom variation, altering venom composition during feeding could be yet another key to the success of this diverse group of marine gastropods.

## Supporting Information

Figure S1 **RP-HPLC chromatograms of 1^st^, 2^nd^, and 3^rd^ injections from a Hawaiian** ***C. textile*** **specimen and** ***C. textile*** **duct venom.** Quantitative differences in venom composition after multiple injections during a single prey capture event of a Hawaiian *C. textile* specimen shown by reverse-phase high performance liquid chromatography (∼50 µls per injection). Chromatograms were normalized to maximum peak height. Duct venom (DV) sampled across the entire length of the venom duct was processed and analyzed in the same manner as the injected venom samples (see methods section).
(TIF)

Table S1 **Comparative analysis of relative peak areas for the RP-HPLC analyses presented in** **figure 3** **.** To compare major peaks (with areas larger than the peak eluting at 19.9 min.) among first and third injections, peaks were normalized to this 19.9 min. eluting peak. Peaks not detected in first or third injections are indicated with fold changes > or < respectively. Peaks containing peptides identified by mass spectrometry are indicated (ID).
(TIF)

Movie S1 **Collection of injected venom samples from** ***Conus textile*** **.** Example of how prey capture was stimulated and injected venom samples were collected for analysis.
(MOV)
