# Supplementary figures and images for: Venom Variation during Prey Capture by the Cone Snail, Conus textile
Source: PLoS One. 2014 Jun 18;9(6):e98991. doi: 10.1371/journal.pone.0098991 (PMC4062396; doi:10.1371/journal.pone.0098991)

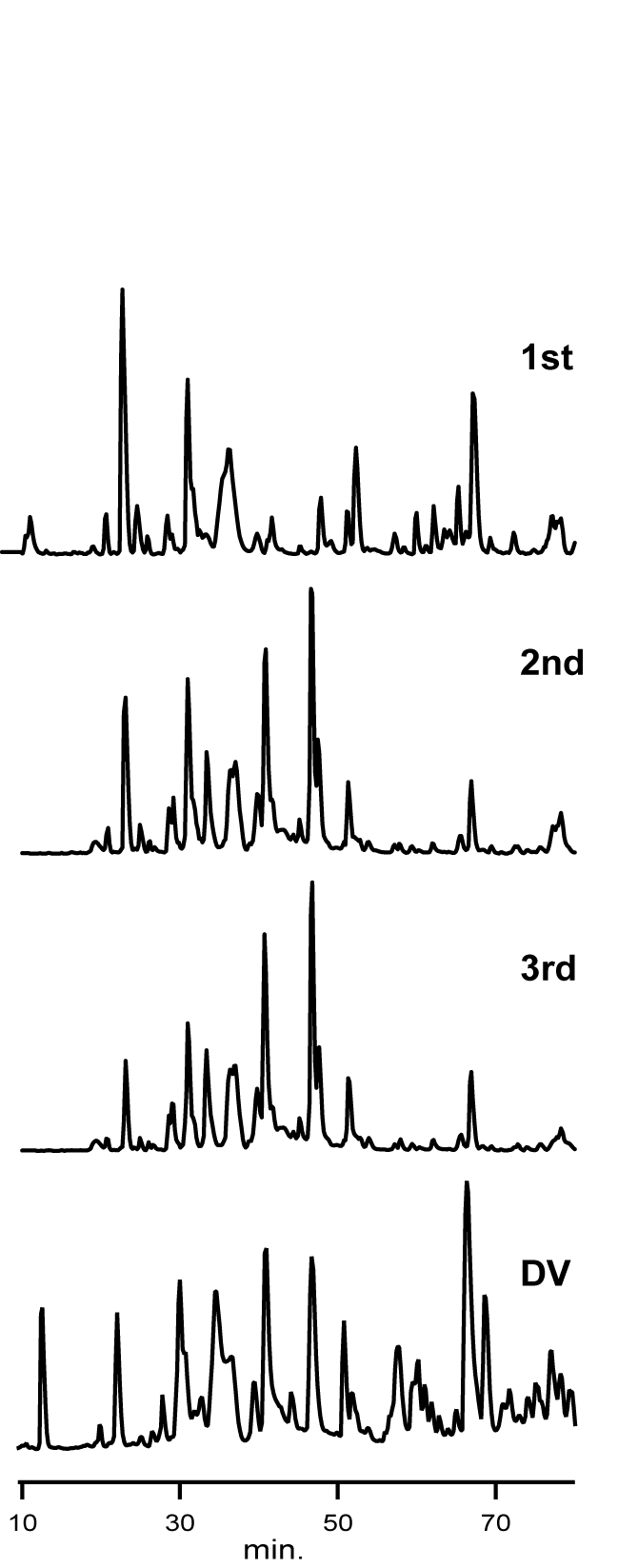

Supplement: Figure S1 — RP-HPLC chromatograms of 1st, 2nd, and 3rd injections from a Hawaiian C. textile specimen and C. textile duct venom. Quantitative differences in venom composition after multiple injections during a single prey capture event of a Hawaiian C. textile specimen shown by reverse-phase high performance liquid chromatography (∼50 µls per injection). Chromatograms were normalized to maximum peak height. Duct venom (DV) sampled across the entire length of the venom duct was processed and analyzed in the same manner as the injected venom samples (see methods section). (TIF) [file pone.0098991.s001.tif]

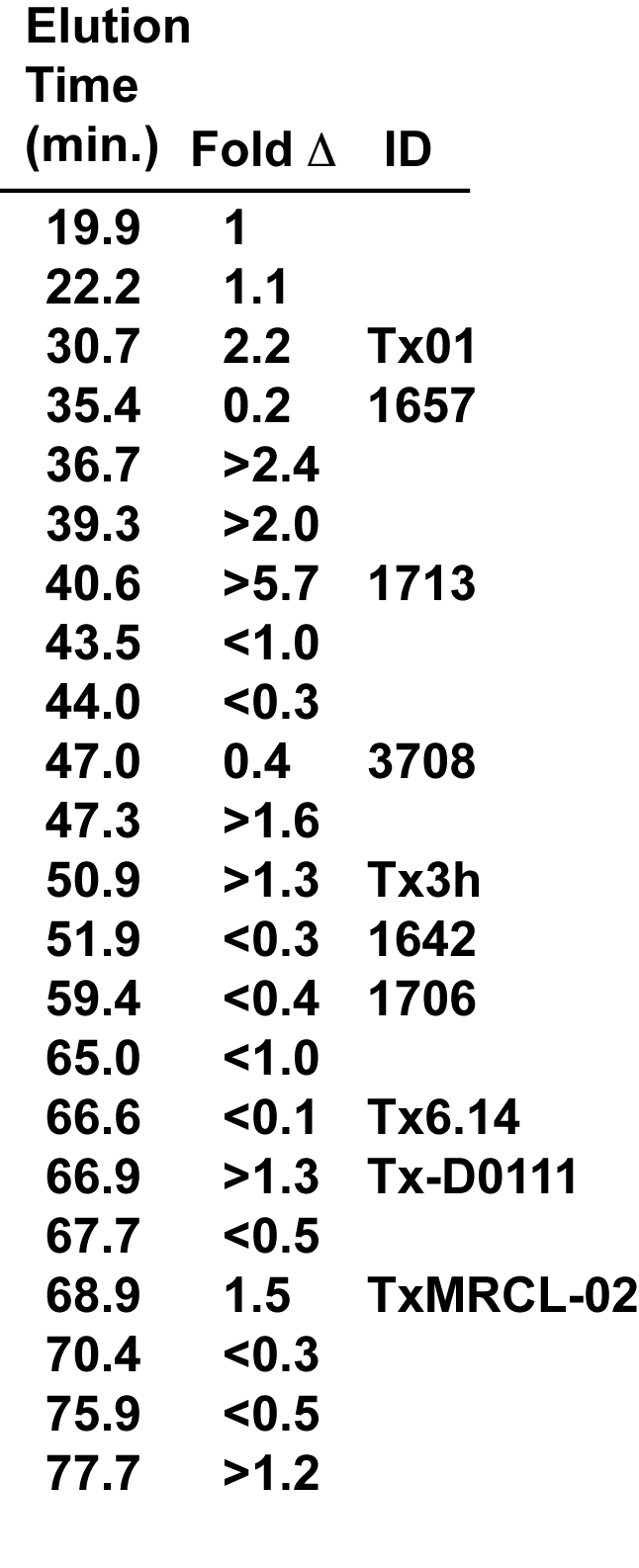

Supplement: Table S1 — Comparative analysis of relative peak areas for the RP-HPLC analyses presented in figure 3 . To compare major peaks (with areas larger than the peak eluting at 19.9 min.) among first and third injections, peaks were normalized to this 19.9 min. eluting peak. Peaks not detected in first or third injections are indicated with fold changes > or < respectively. Peaks containing peptides identified by mass spectrometry are indicated (ID). (TIF) [file pone.0098991.s002.tif]
